# The Mediating Effect of Nurses' Emotional Intelligence in the Relationship between Moral Sensitivity and Communication Ability with Angry Patients

**DOI:** 10.1155/2024/6614034

**Published:** 2024-07-24

**Authors:** Si-Yan Guo, Xiao-Kai Wang, Zhen-Xiang Zhang, Qiu-Jun Zhang, Xue Pan, Cai-Xia Li, Dan-Dan Ke

**Affiliations:** ^1^ School of Nursing and Health Zhengzhou University, Zhengzhou, Henan, China; ^2^ No. 5 Affiliated Hospital of Zhengzhou University, Zhengzhou, Henan, China; ^3^ Zhengzhou Central Hospital Affiliated to Zhengzhou University, Zhengzhou, Henan, China

## Abstract

**Aims:**

To test whether emotional intelligence plays a mediating role in the process by which moral sensitivity affects nurses' ability to communicate with angry patients.

**Background:**

Hospital workplace violence is a global problem that disrupts the normal work order of healthcare, undermines trust between nurses and patients, and threatens the physical and mental health of nurses. Improving nurses' ability to communicate with angry patients to identify and diffuse patients' anger is critical to reducing the nurse-patient conflict and avoiding violence in the hospital workplace.

**Methods:**

The data were collected in China. A sample of 212 nurses completed measures of moral sensitivity, emotional intelligence, and the ability to communicate with angry patients. Structural equation modeling was used to test the study' hypothesis.

**Results:**

Our results suggest that nurses' emotional intelligence mediates the relationship between nurses' moral sensitivity and nurses' ability to communicate with angry patients, with a positive correlation between nurses' moral sensitivity, emotional intelligence, and ability to communicate with angry patients.

**Conclusions:**

The findings showed that nurses' moral sensitivity indirectly influenced nurses' ability to communicate with angry patients by directly influencing emotional intelligence. This study provides a theoretical and methodological approach to mitigate nurse-patient conflict and reduce violence in the hospital workplace through a moral perspective. *Implications for Nursing Management*. Nursing managers should pay attention to the moral sensitivity and emotional intelligence of nurses and promote their moral development and emotional intelligence by strengthening moral education in hospitals, utilizing emotional intelligence training courses and narrative nursing, ultimately promoting nurses' ability to communicate with angry patients, further contributing to the reduction of nurse-patient conflict, avoiding violence in the hospital workplace, building a safer hospital environment, promoting the overall development of nurses, and contributing to the development of global health and wellness.

## 1. Introduction

Workplace violence in hospitals continues to be a global problem that disrupts the normal work order of healthcare, undermines trust between nurses and patients, leads to nurses' emotional exhaustion and resignation, and even threatens the lives of nurses [1–3]. Anger, as an innate human emotion, is a precursor to the deterioration of all kinds of interpersonal relationships [4]. The accumulating anger of patients is a direct factor and trigger for eventual nurse-patient conflict and hospital workplace violence. Therefore, the key to resolving nurse-patient conflict and preventing violence in the hospital workplace lies in timely and accurate identification of patients' anger, understanding the cause of their anger, and effectively communicating with them [5]. Nurses are most closely related to patients and serve as the “outpost” of hospital violence. It has been shown that there is a negative correlation between nurses' communication ability and medical disputes and nurse-patient conflicts [6]. In other words, the higher the communication ability of nurses, the fewer nurse-patient conflicts, and the lower the likelihood of hospital workplace violence [7]. At the same time, the nurse's emotions as an individual also need to be seen when considering the nurse's communication with an angry patient. Nurses who fail to control their emotions often respond to difficult-to-communicate patients with anger and frustration [8]. But nurses with high emotional intelligence can successfully resolve conflicts and contradictions from angry patients [9–11]. Therefore, it can be seen that not only does the nurse's communication ability play a role, but their level of emotional intelligence also influences the outcome of handling nurse-patient conflicts. Research has also proven that nurses' communication ability is related to moral sensitivity [12]. The higher the nurses' moral sensitivity, the greater the ability to detect patients' problems in the clinical environment, and the better the nurses' communication ability [13]. Thus, this issue cannot be viewed in isolation; when considering nurses' ability to communicate with angry patients, we must comprehensively consider both their moral sensitivity and emotional intelligence. This study takes a moral psychology perspective to explore whether moral sensitivity affects nurses' ability to communicate with angry patients through direct or indirect pathways. The findings of this study will provide nursing managers with new perspectives and approaches to resolving the problem of medical conflicts and hospital workplace violence.

## 2. Literature Review and Hypotheses

### 2.1. The Concepts of Moral Sensitivity, Emotional Intelligence, and the Ability to Communicate with Angry Patients

Moral sensitivity is the ability to recognize ethical issues and be aware of the consequences of ethical decisions [14]. Nurses' moral sensitivity is defined as their understanding of patients' vulnerability, their ability to identify potential conflicts, and their capacity to accurately infer patient issues while being aware of the consequences of patient decisions [15]. This enables nurses to make favorable decisions for patients.

Emotional intelligence refers to the ability to recognize, control, and utilize one's own emotions and the emotions of others [16].

The ability to communicate with angry patients refers to nurses' capacity to recognize the patient's anger early, identify the cause of the anger, and diffuse it through effective communication skills [17]. In developing the scale, Chen argued that nurses' ability to communicate with an angry patient encompasses four areas: the nurse's recognition of the patient's anger, exploration of the cause of the patient's anger, the nurse's personal preparedness, and communication skills.

### 2.2. Studies on Moral Sensitivity and Emotional Intelligence

A strong positive correlation exists between the moral reasoning ability of nurse leaders and their emotional intelligence [18]. Similarly, a study pointed out that emotional intelligence is an important variable in ethical decision-making [19]. Further research into the moral sensitivity and emotional intelligence of nurses by Kim found a positive correlation between nurses' emotional intelligence and moral sensitivity in a study conducted among Korean nurses [15]. Emotional intelligence and moral sensitivity play crucial roles in influencing nurses' ethical decision-making [20]. Furthermore, higher levels of moral sensitivity, emotional intelligence, and work engagement in nurses contribute to improved interpersonal relationships [21]. Moral sensitivity and emotional intelligence are recognized as key elements influencing interpersonal relationships.

### 2.3. Studies on Moral Sensitivity and the Ability to Communicate with Angry Patients

The communication of bad information to patients or families poses an ethical challenge, leading to extensive research on moral sensitivity in this context. Studies have demonstrated that nurses' moral sensitivity affects their communication ability with patients and their families [22, 23]. Nurses with high levels of moral sensitivity tend to perform better in delivering adverse events reports to patients and families, engaging in honest and safe communication [24]. A high level of moral sensitivity enables nurses to be more attuned to the physical and psychological conditions of patients and actively seek verbal or nonverbal approaches to problem-solving [25].

### 2.4. Studies on Emotional Intelligence and the Ability to Communicate with Angry Patients

Emotional intelligence is recognized as a vital foundation of nurses' conflict resolution abilities. Studies have demonstrated that emotional intelligence as one important component of nurses' personality traits can predict various coping strategies for dealing with conflict [9, 26]. Moreover, emotional intelligence significantly impacts the frequency of workplace violence experienced by nurses. Research has also revealed that emotional intelligence catalyzes improved communication, effectively addressing workplace violence through enhanced communication skills [10]. Emotional intelligence influences nurses' communication style and determines the quality of patient-nurse interactions. Consequently, researchers recommend enhancing the level of emotional intelligence among nurses to improve the quality and safety of patient care [27].

### 2.5. Conceptual Model

This study utilized the social cognitive theory to establish a conceptual model that examines the relationship between nurses' moral sensitivity, emotional intelligence, and their ability to communicate with angry patients [28]. According to the social cognitive theory, individuals learn and develop their behaviors through continuous interactions between personal factors, environmental factors, and behavioral factors. In this study, nurses' moral sensitivity and emotional intelligence are considered as personal factors, the mood of angry patients as an environmental factor, and nurse-patient communication ability is considered as a behavioral factor. Based on the conceptual model, the following hypotheses were proposed:

1. There is a correlation between nurses' moral sensitivity, emotional intelligence, and ability to communicate with angry patients.
2. Nurses' moral sensitivity can directly and positively influence nurses' emotional intelligence.
3. Nurses' moral sensitivity can directly and positively influence nurses' ability to communicate with angry patients.
4. Nurses' emotional intelligence can directly and positively influence nurses' ability to communicate with angry patients.
5. Nurses' moral sensitivity can indirectly influence nurses' ability to communicate with angry patients by affecting nurses' emotional intelligence.

## 3. Methods

### 3.1. Aims

This study aimed to describe the levels of moral sensitivity, emotional intelligence, and communication ability with angry patients among nurses, and to test whether emotional intelligence plays a mediating role in the process by which moral sensitivity affects nurses' ability to communicate with angry patients.

### 3.2. Design

This study used a cross-sectional research design and correlation analyses to examine the mediating role of emotional intelligence in the relationship between moral sensitivity and nurses' ability to communicate with angry patients. To determine the relationship between these variables, responses from participants were gathered through an online survey. The language used in the questionnaire was Chinese.

### 3.3. Participants

Nurses who participated met the following inclusion criteria: (a) having worked for one year and above in the current hospital; and (b) willing to participate in the study. The minimum sample size for structural equation modeling (SEM) is 200 [29]. According to the statistician's recommendations, 5 to 10 times the maximum number of items in the study scale was calculated to determine the sample size in this range [30, 31]. Using the Monte Carlo method [32] based on simulation techniques, it was determined that the minimum sample size required to achieve the desired level of statistical power (80%) was 90 cases. In this study, we recruited 212 participants. The statistical power level for the sample size of 212 cases obtained in this study was 91%. Exclusion criteria were those who did not complete the scale and selected the same answer for all of them, and those who were away from clinical work for more than three months for various reasons before the start of the study. Nurses participating in the study were drawn from a tertiary public hospital in Zhengzhou, Henan Province, China, using a systematic sampling method.

### 3.4. Variable Measurements

#### 3.4.1. Moral Sensitivity

The Moral Sensitivity Questionnaire was developed by Lützén and revised in 2006 [33]. It was translated and culturally adapted by Huang to make the MSQ-R more appropriate for Chinese nurses [34]. The Chinese version of the MSQ-R consists of two dimensions, Moral Burden (4 items) and Moral Responsibility (5 items), and is scored on a 6-point Likert-type scale ranging from 1 “totally disagree” to 6 “totally agree.” Scores range from 9 to 54, with higher scores indicating higher levels of moral sensitivity, with a total score of <32 indicating low sensitivity, 32–43 indicating moderate sensitivity, and >43 indicating high sensitivity. The Cronbach's *α* of this scale in this study is 0.896.

#### 3.4.2. Emotional Intelligence

The Emotional Intelligence Scale was developed in 2002 [35]. In 2010, Wang translated the scale into Chinese and administered it to university students, civil servants, and corporate employees using the Chinese version. The scale consists of four dimensions: Self-Emotional Assessment (4 items), Emotional Assessment of Others (4 items), Emotional Control (4 items), and Emotional Application (4 items). Using a 7-point Likert scale (1 = “I totally disagree” to 7 = “I totally agree”), the scale scores range from 7 to 112, with higher scores indicating higher emotional intelligence. The Cronbach's *α* of this scale in this study is 0.964.

#### 3.4.3. Nurse's Communication Ability with Angry Patients Scale (NCAAPS)

Developed by Chen in 2021 in China, the NCAAPS consists of 4 dimensions: anger perception (3 items), cause exploration (6 items), self-preparation (7 items), and communication skills (3 items) [17]. The options were assigned from “Strongly Disagree” to “Strongly Agree” on a scale of 1–5, with higher scores indicating better communication skills in angry situations, with a maximum score of 95. The Cronbach's *α* of this scale in this study is 0.934.

### 3.5. Data Analysis

The data was analysed using SPSS and AMOS software. First, SPSS was used to filter and remove invalid questionnaires. The total number of participants who chose the same answers for all questions was five, and the total number of participants who did not fill in some of the answers was three. These invalid questionnaires were removed as recommended by existing studies [36]. Descriptive statistics were used to describe general characteristics. Relationships between all dimensions were assessed by Pearson correlation analysis. Validating factor analysis was used to test the applicability of the AMOS measurement model, followed by structural equation modeling to verify the relationship between moral sensitivity, emotional intelligence, and nurses' ability to communicate with angry patients. The mediating role of emotional intelligence was also tested.

### 3.6. Ethical Considerations

Before the investigation, the study was approved by the university ethics committee (approval number: 2023-109) and by the hospital nursing department. Nurses volunteered to participate in this study and could quit at any time. The electronic questionnaire was completed anonymously.

## 4. Results

### 4.1. Participants' Sociodemographic Characteristics

The demographic characteristics of the participants are shown in Table 1. The majority of participants were female (69.3%) and married (80.2%). The mean age of the participants was 35.35 years (SD = 7.51) and the mean number of years worked was 14.82 years (SD = 9.26). The general information characteristics of the sample drawn were generally consistent with those of previously studied Chinese nurses [37].

### 4.2. Moral Sensitivity, Emotional Intelligence, and Nurses' Ability to Communicate with Angry Patients and the Association between the Variables

Descriptive statistics for the key variables are shown in Table 2, with a total mean score of 46.62 (SD = 7.55) for moral sensitivity, 92.92 (SD = 15.42) for emotional intelligence, and 81.64 (SD = 11.62) for the nurses' ability to communicate with angry patients. As shown in the correlation matrix in Table 2, moral sensitivity was significantly positively correlated with emotional intelligence (*r* = 0.544, *p* < 0.001) and also with nurses' ability to communicate with angry patients (*r* = 0.734, *p* < 0.001). The nurses' emotional intelligence was also significantly positively correlated with nurses' ability to communicate with angry patients (*r* = 0.627, *p* < 0.001).

### 4.3. Structural Equation Model

**Figure 1:**
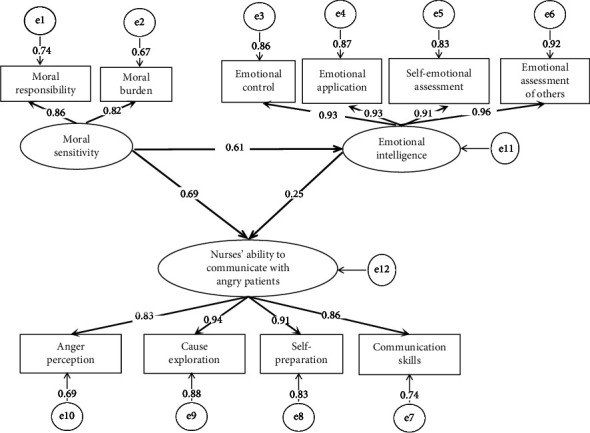
Standardized coefficients for path analysis of direct and indirect effects of moral sensitivity on the ability of nurses to communicate with angry patients mediated by nurses' emotional intelligence.

Using moral sensitivity as the independent variable, the nurses' ability to communicate with angry patients as the dependent variable, and emotional intelligence as the mediating variable, a structural equation model plot was created using AMOS. The model fit indices *X*^2^/df = 2.125 (<3), RMSEA = 0.06 (<0.08), CFI = 0.986(>0.9), IFI = 0.986 (>0.9), GFI = 0.943 (>0.9), and TLI = 0.981 (>0.9), were all within acceptable limits, indicating that the structural model fitted well (Figure 1).

### 4.4. The Mediating Effect Analysis

As shown in Table 3, moral sensitivity has a direct positive effect on both emotional intelligence (*β* = 0.61, *p* < 0.001) and the nurse's ability to communicate with angry patients (*β* = 0.69, *p* < 0.001). Emotional intelligence directly and positively influenced nurses' ability to communicate with angry patients (*β* = 0.25, *p* < 0.001). Moral sensitivity also positively influences nurses' communication skills with angry patients indirectly through emotional intelligence (*β* = 0.15, *p* < 0.001). The findings suggest that nurses' moral sensitivity indirectly affects nurses' ability to communicate with angry patients by directly influencing nurses' emotional intelligence. Emotional intelligence played a mediating role in the influence of moral sensitivity on nurses' ability to communicate with angry patients.

## 5. Discussion

Social cognitive theory suggests that human activity is determined by the interaction of three factors: the external environment in which the individual lives, individual cognition and other individual characteristics, and individual behavior [28]. When a person is placed in an environment, not only does the environment have an impact on the individual's behavior, but the individual's cognition and individual characteristics also play a significant role in the individual's behavior. Based on the background of social cognitive theory, this study aims to examine and clarify the relationship between nurses' moral sensitivity, emotional intelligence, and their ability to communicate with angry patients. Additionally, it seeks to explore the mechanisms through which nurses' moral sensitivity influences their ability to communicate with angry patients. The main finding of this study highlights the significant mediating role of nurses' emotional intelligence between their moral sensitivity and their ability to communicate with angry patients. Moreover, a strong positive correlation is observed between nurses' emotional intelligence and their moral sensitivity. These findings are consistent with hypotheses based on social cognitive theory.

### 5.1. Moral Sensitivity Influences Nurses' Ability to Communicate with Angry Patients through the Mediating Role of Emotional Intelligence

#### 5.1.1. Moral Sensitivity Directly Affects Nurses' Ability to Communicate with Angry Patients

This study discovered a significant positive correlation between nurses' moral sensitivity and their ability to communicate with angry patients. The findings of this study indicate that higher levels of moral sensitivity in nurses are associated with better communication abilities when interacting with angry patients and a reduced likelihood of nurse-patient conflict. These findings align with previous studies that have also highlighted the relationship between nurses' communication abilities and moral sensitivity [26, 38].

This study further revealed that nurses with a heightened awareness of their moral responsibility exhibited better assessment of the emotional state of angry patients, their own emotions, emotional preparedness, and emotional control (Table 2). Additionally, nurses with high levels of moral sensitivity demonstrated increased sensitivity to potential nurse-patient conflicts in clinical settings, enabling them to detect the accumulation of anger in patients more effectively and promptly (Table 2). Furthermore, nurses with high moral sensitivity displayed a greater awareness of their moral responsibilities and burdens, leading them to proactively address communication issues with angry patients (Table 2).

#### 5.1.2. Emotional Intelligence Mediates the Relationship between Moral Sensitivity and the Nurses' Ability to Communicate with Angry Patients

We can find that from this study, moral sensitivity has a positive impact on nurses' ability to communicate with angry patients, and emotional intelligence plays a mediating role between these two factors; nurses' moral sensitivity indirectly influences their ability to communicate with angry patients by directly influencing their emotional intelligence.

Nurses with high moral sensitivity are adept at identifying moral issues by gathering information from their environment. When they become aware of ethical dilemmas, their emotional intelligence is activated, allowing them to integrate their own emotions with those of others, ultimately leading to problem-solving behaviors. Moral sensitivity provides cognitive material and cues, while emotional intelligence integrates them. Through the use of emotional intelligence, nurses can identify patients' anger and mobilize their emotional intelligence to explore the underlying causes, evoke positive emotions within themselves, and employ effective communication skills to defuse the patients' anger. In this way, nurses' emotional intelligence serves as a bridge between moral sensitivity and their ability to communicate with angry patients.

### 5.2. Practical Implications

#### 5.2.1. Promotions of Nurses' Moral Sensitivity

Reflecting on existing research, the overarching goal of studies on nurse ethics and nurse ethics education is to foster nurses' moral consciousness. This aims to ensure that nurses adhere to ethical principles in clinical practice and make decisions that maximize patient benefits in complex healthcare scenarios. The cultivation of nurses' moral sensitivity is crucial in this regard.

To enhance nurses' moral sensitivity, managers can establish a moral education platform for nurses through interdisciplinary collaboration, including integration with humanities and social science disciplines. This integration serves to promote nurses' moral sensitivity and incorporate it into their professional practice. A panel consisting of nursing researchers, humanities and social science researchers, and other relevant stakeholders can be convened to determine the course content based on existing literature on moral sensitivity in nursing and theoretical models of moral sensitivity.

The course can be structured in two phases. In the first phase, nurses receive lectures on the theoretical aspects of the moral sensitivity course. The second phase adopts a discussion and questioning approach, encouraging nurses to raise questions and engage in moral discussions regarding potential issues that may arise in clinical settings. This interactive approach encourages nurses to actively explore and analyse moral dilemmas, thereby further developing their moral sensitivity [39]. Kant, in his work “On Pedagogy,” emphasizes that the ultimate goal of education is to enable individuals to transition from “heteronomy” to “self-discipline” [40, 41]. In other words, the ideal moral development of nurses entails the transformation of external moral constraints into internal moral principles. Successful moral education for nurses empowers them to transcend their previous moral selves and attain a higher level of moral sensitivity. In complex clinical settings, nurses can utilize their moral sensitivity to identify potential patient issues and engage in altruistic behavior instinctively. This is because nurses have internalized morality, enabling them to act based on self-discipline rather than external constraints.

#### 5.2.2. Improvements of Nurses' Emotional Intelligence

Nursing managers should indeed prioritize the development of nurses' emotional intelligence, as nurses with poor emotional regulation may struggle with complex moral choices and experience heightened negative emotions in the face of ongoing moral stress. To address this, training courses on emotional intelligence can be conducted to provide nurses with education on emotional recognition, emotional control, and communication skills. These courses should aim to enhance nurses' understanding of emotional intelligence by explaining its theories and theoretical models.

The course also can be designed to improve nurses' emotional intelligence through interactive activities such as self-reporting, role-playing, and group debriefing. Nurses can be randomly divided into small groups and assigned various clinical cases for discussion. Each group can then use a PowerPoint presentation to analyse the emotional aspects of the case, including the emotions of both the nurse and the patient, identify any inappropriate emotional responses from the nurse, and suggest measures to address the case. Incorporating emotional intelligence assessment into the evaluation criteria for nurses' abilities and performance is also recommended.

Furthermore, nursing managers can consider utilizing narrative medicine and narrative care to enhance nurses' emotional intelligence. These approaches involve utilizing storytelling and reflective practices to promote self-awareness and empathy, thereby improving emotional intelligence. Establishing relevant departments within hospitals to provide support and assistance in enhancing nurses' emotional intelligence can further contribute to their development in this area.

#### 5.2.3. Training on Nurses' Ability to Communicate with Angry Patients, Reduce the Occurrence of Workplace Violence

To train nurses on communicating emotions with angry patients, firstly, nurses must learn to identify patients' anger in a timely and accurate manner. They can improve their ability to identify patients' anger by reading relevant books, pictures and videos, and sharing good communicational cases. Nursing managers can also incorporate existing research, such as psychological sociology, to develop models of patients' anger changes and set up relevant training and courses in hospitals to improve nurses' ability to communicate with angry patients.

## 6. Limitations

This study explored the relationship between nurses' moral sensitivity, emotional intelligence, and ability to communicate with angry patients. However, this study sampled some nurses in China and the findings may not be completely representative of other countries. Future researchers will need to further expand the sample participants for the study. Additional variables could be considered for future studies to explore the role that other variables play in the relationship between nurses' moral sensitivity, emotional intelligence, and ability to communicate with angry patients.

## 7. Conclusions

This study aimed to explore the relationship between nurses' moral sensitivity, emotional intelligence, and ability to communicate with angry patients. The findings showed that there was a positive relationship between nurses' moral sensitivity, emotional intelligence, and ability to communicate with angry patients, and that emotional intelligence mediated the relationship between moral sensitivity and ability to communicate with angry patients. Nurses' moral sensitivity indirectly influenced nurses' ability to communicate with angry patients by directly influencing emotional intelligence. This study provides new perspectives and methods for alleviating nurse-patient conflict and resolving hospital violence [42].

## Figures and Tables

**Table 1:** Sociodemographic characteristics of participants (*n* = 212).

Nurse characteristics	Mean (SD)	*N* (%)
Gender		
Male		65 (30.7)
Female		147 (69.3)
Age	35.35 (7.51)	
20∼30		62 (29.2)
31∼40		81 (38.2)
>40		69 (32.6)
Years in nursing	14.82 (9.26)	
1∼5		47 (22.2)
6∼10		47 (22.2)
11∼20		64 (30.2)
>20		54 (25.4)
Professional title		
Junior		94 (44.3)
Intermediate		87 (41.1)
Senior		31 (14.6)
Education level		
Secondary vocational		18 (8.5)
Associate degree		57 (26.9)
Bachelor's degree		126 (59.4)
Master's degree or above		11 (5.2)
Marital status		
Married		170 (80.2)
Single		39 (18.4)
Other (divorce/widowed)		3 (1.4)

**Table 2:** Statistical description and correlation matrix results for study variables (*n* = 212).

	M (SD)	Cronbach' *α*		MR	MB	MS	AP	CE	SP	CS	NCAAP	EC	EA	SA	EAO	EI
MR	5.36 (0.88)	0.972	r	1												
MB	4.94 (0.98)		r	0.699^*∗∗*^												
MS	46.62 (7.55)		r	0.919^*∗∗*^	0.923^*∗∗*^											

AP	4.29 (0.66)	0.975	r	0.614^*∗∗*^	0.543^*∗∗*^	0.628^*∗∗*^										
CE	4.25 (0.69)		r	0.692^*∗∗*^	0.661^*∗∗*^	0.730^*∗∗*^	0.787^*∗∗*^									
SP	4.3 (0.66)		r	0.633^*∗∗*^	0.564^*∗∗*^	0.646^*∗∗*^	0.732^*∗∗*^	0.840^*∗∗*^								
CS	4.34 (0.67)		r	0.617^*∗∗*^	0.598^*∗∗*^	0.656^*∗∗*^	0.693^*∗∗*^	0.799^*∗∗*^	0.833^*∗∗*^							
NCAAP	81.64 (11.62)		r	0.703^*∗∗*^	0.655^*∗∗*^	0.734^*∗∗*^	0.865^*∗∗*^	0.942^*∗∗*^	0.940^*∗∗*^	0.903^*∗∗*^						

EC	5.75 (1.06)	0.968	r	0.473^*∗∗*^	0.454^*∗∗*^	0.502^*∗∗*^	0.494^*∗∗*^	0.601^*∗∗*^	0.583^*∗∗*^	0.499^*∗∗*^	0.601^*∗∗*^					
EA	5.8 (0.98)		r	0.482^*∗∗*^	0.497^*∗∗*^	0.530	0.503^*∗∗*^	0.586^*∗∗*^	0.520^*∗∗*^	0.474^*∗∗*^	0.574^*∗∗*^	0.859^*∗∗*^				
SA	5.88 (0.95)		r	0.451^*∗∗*^	0.473^*∗∗*^	0.502^*∗∗*^	0.488^*∗∗*^	0.582^*∗∗*^	0.571^*∗∗*^	0.527^*∗∗*^	0.597^*∗∗*^	0.856^*∗∗*^	0.821^*∗∗*^			
EAO	5.8 (0.97)		r	0.482^*∗∗*^	0.488^*∗∗*^	0.527^*∗∗*^	0.510^*∗∗*^	0.608^*∗∗*^	0.561^*∗∗*^	0.507^*∗∗*^	0.602^*∗∗*^	0.870^*∗∗*^	0.896^*∗∗*^	0.870^*∗∗*^		
EI	92.92 (15.42)		r	0.498^*∗∗*^	0.504^*∗∗*^	0.544^*∗∗*^	0.527^*∗∗*^	0.628^*∗∗*^	0.590^*∗∗*^	0.530^*∗∗*^	0.627^*∗∗*^	0.949^*∗∗*^	0.943^*∗∗*^	0.936^*∗∗*^	0.959^*∗∗*^	1

r: Pearson coefficient; ^*∗∗*^Statistically significant at *p* < 0.001; MR: moral responsibility; MB: moral burden; MS: moral sensitivity; EC: emotional control; EA: emotional application; SA: self-emotional assessment; EAO: emotional assessment of others; EI: emotional intelligence; AP: anger perception; CE: cause exploration; SP: self-preparation; CS: communication skills; NCAAP: nurse's communication ability with angry patients.

**Table 3:** Mediating effect.

	*β*	95%CI		*p*
Direct effect of MS on NCAAP	0.69	0.50	0.84	<0.001
Effect of MS on EI	0.61	0.45	0.73	<0.001
Effect of EI on NCAAP	0.25	0.09	0.43	<0.001
Indirect effect	0.15	0.05	0.28	<0.001
Total effects	0.84	0.74	0.91	<0.001

MS: moral sensitivity; EI: emotional intelligence; NCAAP: Nurse's Communication Ability with Angry Patients.

## Data Availability

The datasets generated during and/or analysed during the current study are available from the corresponding author on reasonable request.
